# The Effect of Aging Time at 600 °C on Tensile Properties of the 0.3Nb FeCrAl Alloy

**DOI:** 10.3390/ma18071684

**Published:** 2025-04-07

**Authors:** Liping Tang, Hongying Sun, Guijun Wu, Zhangquan Lv, Yi Xiong

**Affiliations:** 1 School of Mechanical and Aeronautical Manufacturing Engineering, Anyang Institute of Technology, Anyang 455000, China; tangliping57@163.com (L.T.); 20160277@ayit.edu.cn (G.W.); lvzq2016@163.com (Z.L.); 2 School of Materials Science and Engineering, Henan University of Science Technology, Luoyang 471023, China; 3 Department of Mechanics Engineering, Kyrgyz State Technical University named after I. Razzakov, Aitmatov av., 66, Bishkek 720044, Kyrgyzstan

**Keywords:** aging, tensile properties, FeCrAl alloy, tensile properties

## Abstract

This study examines the impact of aging at 600 °C on the tensile properties of 0.3NbFeCrAl alloy at various temperatures, including room temperature (RT), 300 °C, 350 °C, and 400 °C, as well as the corresponding changes in microstructure. Results demonstrate that as aging time increases, the grain size remains relatively stable, while the amount of precipitate gradually increases and becomes uniformly distributed. The tensile strength (R_m_) also increases steadily with aging time, reaching its maximum after 1000 h of aging. This can be attributed to the precipitation strengthening effect of the Laves phase after 1000 h of aging. The yield strength (R_p0.2_) remains constant when the specimen is stretched at room temperature, but gradually increases with both the stretching temperature and aging time. Additionally, the section shrinkage ratio (Z) exhibits a decreasing trend with aging time, except for stretching at room temperature. Nevertheless, Z remains above 50% in all other cases, indicating that the toughness of the aged specimens is maintained well. While fracture shrinkage is significant for tensile tests at RT, it exhibits minimal change with increasing aging time. Furthermore, a notable increase in the number of dimples and a decrease in their size is observed on the tensile fracture surface with aging.

## 1. Introduction

Due to its excellent high-temperature steam oxidation resistance, radiation resistance, and corrosion resistance, FeCrAl alloy has become one of the most important candidates to replace Zr-based alloy as advanced accident tolerance fuel (ATF) cladding materials in light water reactor (LWRS) applications [1,2,3,4]. However, the subpar mechanical properties of pure FeCrAl alloy limit its application in LWRS. To meet the requirements of nuclear energy and improve its mechanical properties, extensive research has been conducted on FeCrAl alloy. Fang X Q et al. studied the microstructure evolution and hot deformation behavior of FeCrAl alloy during the rolling process. The hot working diagram was established and the optimum hot working range was given. The optimum working range of the material is under the true strain of 0.69, the deformation temperature should be 1050–1200 °C, and the strain rate should be 0–0.01 s^−1^ [5]. Ding et al. prepared a ZrC dispersion-reinforced FeCrAl alloy using spark plasma sintering and hot forging, resulting in a material with improved high-temperature strength and plasticity. Their study demonstrated that the addition of a small amount of ZrC nanoparticles significantly refined the grain structure and enhanced strength and hardness of the FeCrAl alloy [6]. Han L et al. investigated the effects of nanoparticles containing carbide (ZrC) and oxide (Y_2_O_3_) on the microstructure and mechanical properties of FeCrAl alloys. It was found that Zr preferentially reacted with C to form ZrC rather than Y-Zr-O particles, and the alloyed material with the addition of Y_2_O_3_ formed oxides with finer particle sizes. Samples containing only Y_2_O_3_ exhibited the best tensile properties [7]. Ma Z et al. synthesized a nano-ZrC reinforced FeCrAl alloy by mechanical alloying and spark plasma sintering and studied the influence of nano-ZrC on the mechanical properties and electrochemical corrosion properties of FeCrAl alloy. Their findings indicated that the appropriate addition of nano-ZrC could effectively improve the mechanical properties and corrosion properties of FeCrAl alloy [8]. Zhang Y et al. studied the crystal structure, microstructure, tensile, and oxidation resistance to high-temperature steam of FeCrAl alloys with different Cr contents. The study revealed that when the Cr content was below 9 wt.%, minor changes in Cr content had negligible effects on the tensile strength and oxidation resistance of the alloy [9]. In another study, Qu H J et al. studied the oxidation behavior of Fe17Cr5.5Al with different Ni contents in oxygenated and hydrogenated simulated reactors, and the results showed that the addition of Ni improved the passivating of FeCrAl alloy under oxidation conditions and reduced the mass loss under oxidation conditions [10]. Wang H et al. studied the influence of cold rolling reduction on the microstructure and tensile properties of low Cr and low Nb annealed FeCrAl alloys. It was observed that reducing the Cr content by 30–50% with a low Nb concentration of 0.5 wt.% resulted in a stable precipitate size, effectively enhancing the alloy’s strength and yielding the best tensile properties [11]. Sun Z et al. examined the influence of the addition of Mo and Nb on the microstructure and mechanical properties of FeCrAl alloy. Their findings demonstrated that the addition of two trace elements could obtain fine grain structure, and the deformation and recrystallization structure of FeCrAl alloy containing Nb were stable due to the pinning effect of the Laves phase [12]. A H W et al. studied the strengthening mechanism of Nb in FeCrAl alloy, which showed that Nb could precipitate high-density Fe_2_Nb in the alloy, and the alloy showed high tensile strength and reasonable ductility during the test process [13]. Sun Zhiqian et al. investigated the microstructure and mechanical properties of Nb-containing FeCrAl alloy after deformation and annealing, highlighting the stabilizing influence of the Fe_2_Nb-type Laves phase on the alloy’s microstructure [14]. Zheng J et al. investigated the effect of thermo-machining on the microstructure and precipitation behavior of hot-rolled FeCrAl alloy plates. The results showed that after hot rolling, FeCrAl alloy plates had a typical deformation texture. TEM identification results showed that the Laves precipitates were of Fe_2_Nb. After long-term aging at 800 °C, the specimens with uniform Laves phase particles exhibited good thermal stability [15].

Therefore, the incorporation of the Nb element into FeCrAl alloy serves to stabilize the microstructure and enhance the mechanical properties of the alloy. As the nuclear accident-resistant cladding material needs to work in high-temperature environment for a long time, special attention should be paid to the effect of aging in high-temperature environment on the properties of FeCrAl alloy. In a study conducted by Rajendran R et al., the impact of aging and α’ phase precipitation on the oxidation and electrochemical performance of FeCrAl alloy at elevated temperatures was investigated. The findings revealed that the aging process resulted in the formation of a thinner oxide layer and increased corrosion resistance within the aging specimen [16]. Additionally, Zhang Y et al. examined the tensile properties and zigzag plastic flow of Fe-13Cr-4Al alloy under different strain rates ranging from room temperature to 600 °C. The results indicated that as the strain rate increased, the temperature range for the occurrence of zigzag plastic flow shifted towards higher temperatures. Within this temperature range, the alloy exhibited zigzag plastic flow while maintaining stable yield strength and strain-hardening behavior [17]. Furthermore, Li N et al. studied the growth kinetics, chemical composition, and microstructure of oxidation formation of FeCrAl alloy after aging in air at 300, 400, 500, and 600 °C for 100–1000 h. The findings revealed that when the oxidation temperature was below 400 °C, the oxide layer formed was amorphous. However, at 600 °C, the oxidation film exhibited enhanced resistance to oxidation [18]. Current research on FeCrAl alloy aging predominantly focuses on changes in microstructure and oxidation resistance of the oxide layer, with minimal emphasis on the long-term aging effects on the mechanical properties and microstructure of the alloy. To address this research gap, a 0.3Nb FeCrAl alloy was prepared using the vacuum induction furnace method. The mechanical properties of the FeCrAl alloy were investigated by subjecting it to aging at 600 °C and different tensile temperatures.

## 2. Materials and Methods

**Figure 1:**
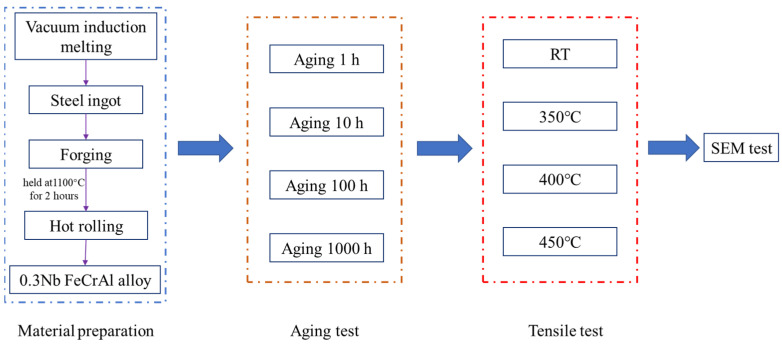
The entire experimental process.

This article explored the preparation of 0.3Nb FeCrAl alloy using the vacuum induction furnace (ZG-0025, Jinzhou ZhongZhen Technology Co., Ltd., Dajin Road, Kaoshan Industrial Zone, Linghai City, Jinzhou, China). The composition of the alloy is provided in Table 1. The theoretical melting point of 0.3 Nb FeCrAl alloy calculated with JMatPro 7 is 1505 °C, and the actual melting temperature is around 1530 °C. The process began by cooling the molten steel to approximately 1200 °C before pouring it into a mold to form a steel ingot. Subsequently, the ingot was forged into a rectangular shape and held at a temperature of 1100 °C for 2 h prior to the first rolling step, during which a 16 mm steel plate was obtained. Following this, the steel plate was cooled to 850 °C for the second rolling, resulting in a 12.5 mm steel plate. The FeCrAl alloy sample was then subjected to a heat treatment process, wherein it was heated to approximately 1150 °C and held at this temperature for 1.5 h. Subsequently, rapid cooling was implemented to achieve supersaturated solid solution characteristics. In order to investigate the aging behavior of the alloy, the specimens were exposed to different aging durations at a temperature of 600 °C. Specifically, aging durations of 1, 10, 100, and 1000 h, respectively, were considered. The entire experimental process is depicted in Figure 1.

**Figure 2:**
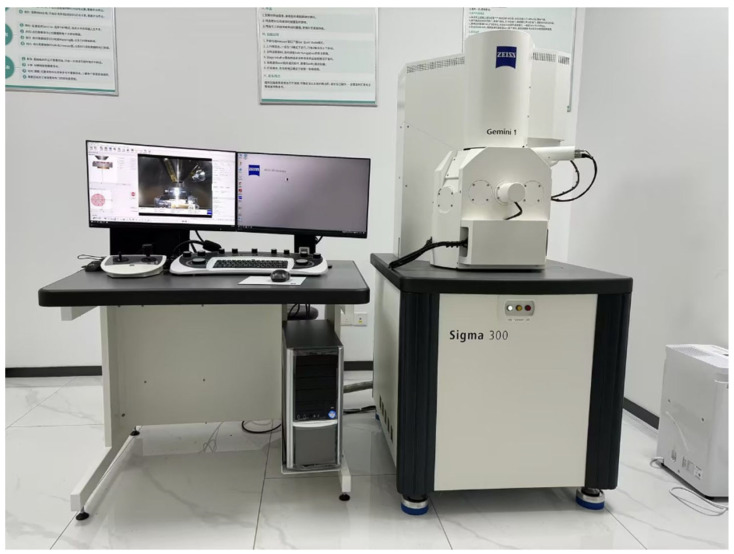
Scanning electron microscopy.

The microstructures of the FeCrAl alloy were assessed using an X-ray diffractometer (XRD, RIGAKU D/MAX-2500, Rigaku Corporation, Tokyo, Japan). The XRD measurement was conducted at a scanning rate of 1°/min, with a step size of 0.013°. In order to examine the surface and cross-sectional morphology of the alloy, scanning electron microscopy (SEM, Zeiss Gemini Sigma 300 VP, Carl Zeiss AG, Oberkochen, Germany) and energy spectroscopy analyzer (EDS, Ultim Max, Hertfordshire, UK) were employed, as shown in Figure 2.

**Figure 3:**
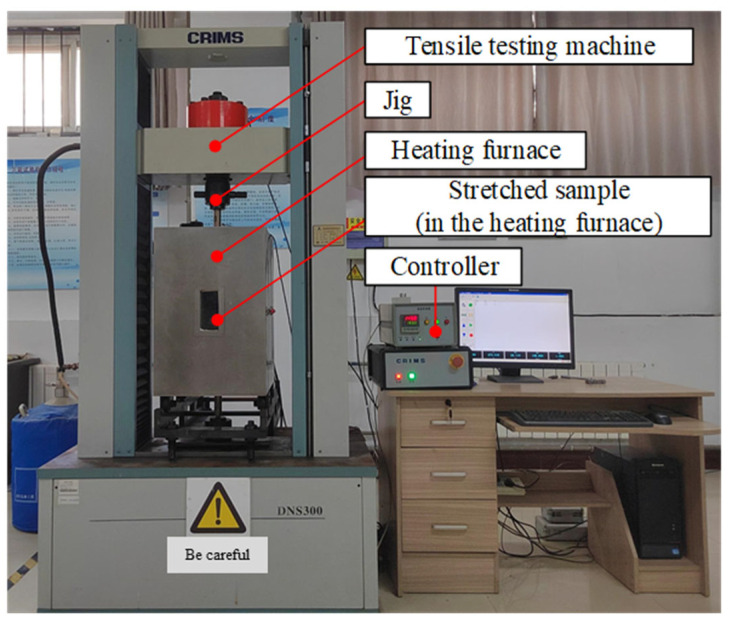
High-temperature tensile test device.

**Figure 4:**
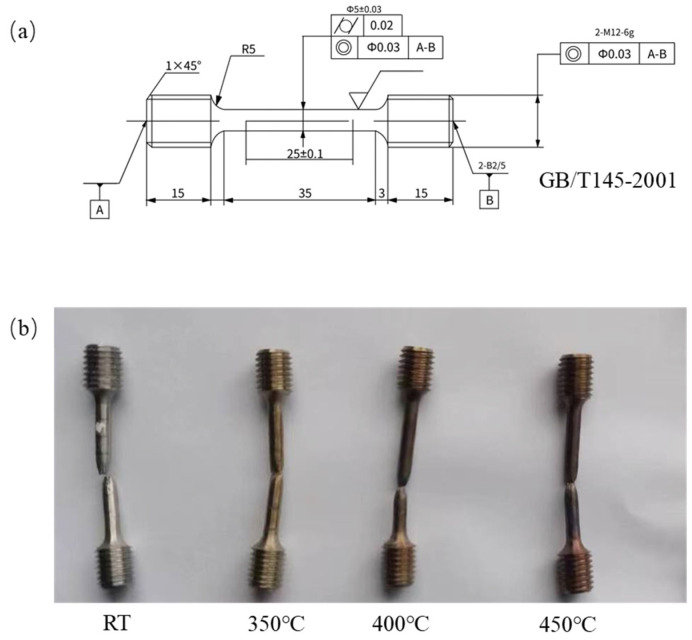
FeCrAl alloy tensile specimen. (**a**) Structure of tensile specimen and requirements for sample preparation [19], (**b**) fracture specimens.

The high-temperature tensile test device (CRIMS DNS300, Changchun Research Institute for Mechanical Science Co., Ltd., 1118 Silicon Valley Street, Changchun, China) was used as shown in Figure 3. It includes a tensile testing machine and a heating furnace. The tensile tests were performed at varying temperatures: room temperature (RT), 350 °C, 400 °C, and 450 °C. A displacement rate of 0.2 mm/min was applied during the tests. Tensile specimens with a gauge length of 25 mm and a diameter of 5 mm were prepared. The structure of the tensile specimen and requirements for sample preparation are shown in Figure 4a. The samples were first heated to the designated testing temperature and held for 20 min prior to the tensile tests. Tensile fracture specimens are shown in Figure 4b. The tensile strength (R_m_), specified plastic extension strength (R_p0.2_), elongation after fracture (A), and section shrinkage (Z) were then determined. Vickers hardness was also measured using a microhardness tester (HV-1000A, Laizhou HuaYin TEST Instrument Co., Ltd., 2788 East Outer Ring Road, Laizhou, China), with each result being an average of four measurements.

## 3. Results and Discussion

### 3.1. Microstructural Evolution During Aging

**Figure 5:**
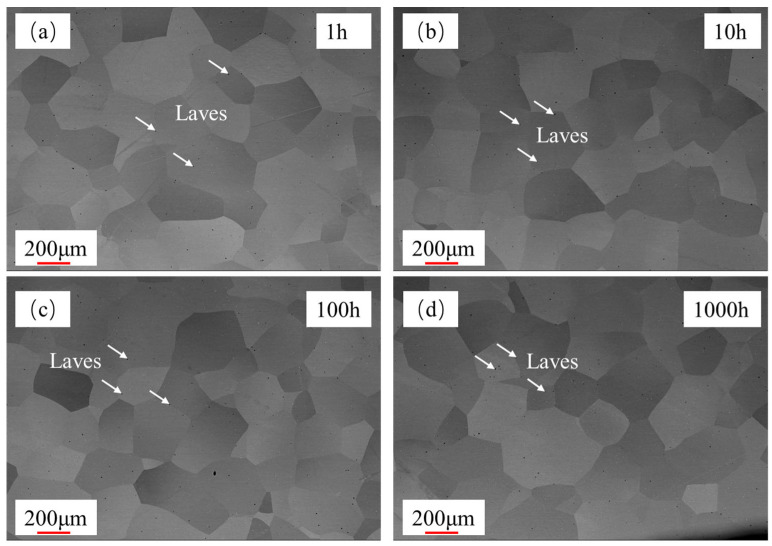
Change in grain size with aging time: (**a**) 1 h, (**b**) 10 h, (**c**) 100 h, and (**d**) 1000 h.

**Figure 6:**
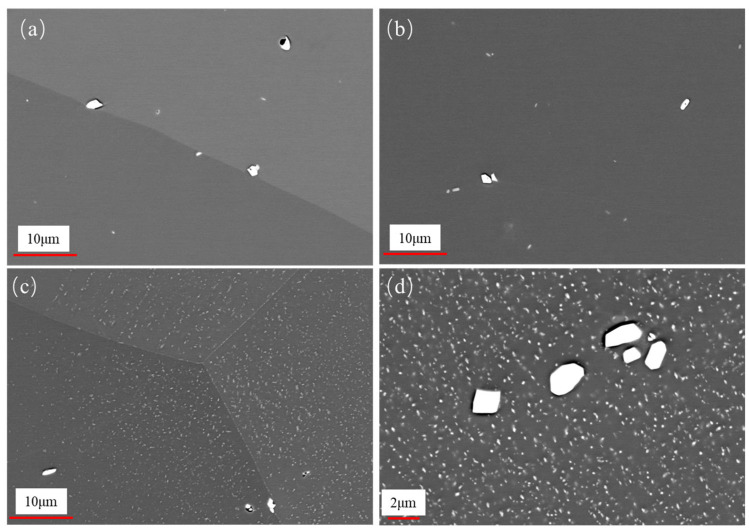
High-magnification image for aged specimens: (**a**) 1 h, (**b**) 10 h, (**c**) 100 h, (**d**) 1000 h.

**Figure 7:**
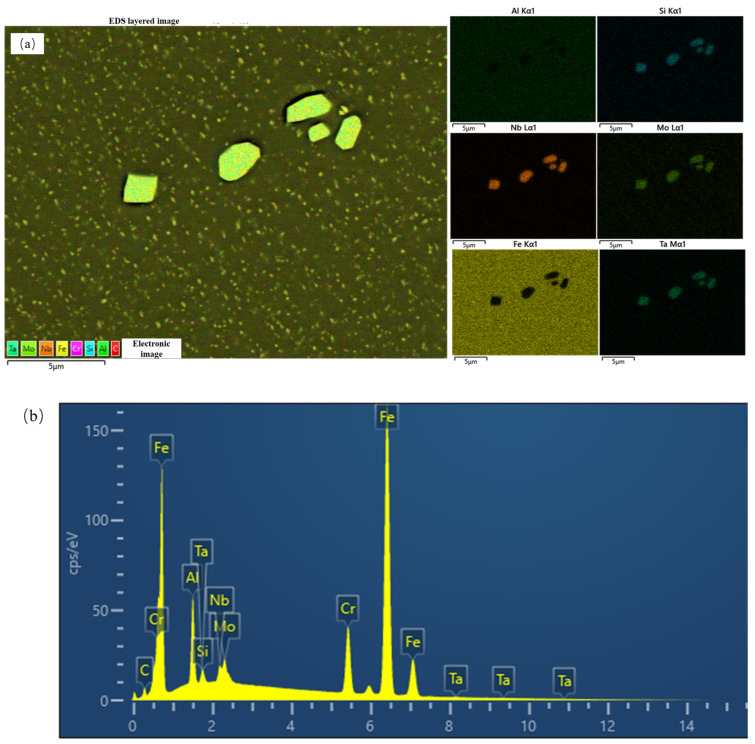
Fe_2_Nb in aged specimen for 1000 h. (**a**) FeCrAl alloy element surface distribution diagram. (**b**) EDS result of precipitate indicated in (**a**).

The morphology of the FeCrAl alloy during aging was investigated. Figure 5 illustrates the temporal changes in grain size of the alloy. The grain size was measured by the cross-section method. The grain sizes were 238.9 μm, 240.0 μm, 215.9 μm, and 224.6 μm after aging for 1 h, 10 h, 100 h, and 1000 h, respectively. It was observed that there is no significant change in grain size within the aging period of up to 1000 h. However, after aging, the metallographic surface presents black spots, which might be attributed to the formation of precipitates; the high-magnification image is shown in Figure 6. From the EDS results of the base material (Figure 7), it can be seen that the precipitate is Fe_2_Nb. The emergence of the Laves phase can be attributed to the high-temperature exposure combined with rapid cooling experienced by the FeCrAl alloy during its processing. Such conditions might facilitate the elimination of internal stresses within the material and enhance its microstructure. As can be seen from Figure 6, with the increase in aging time, the precipitate gradually changes from irregular to elliptical granular; the size after 1000 h of aging is about 2.2 μm for the long axis and 1.3 μm for the short axis. When the precipitate forms a stable structure on the grain boundary, the energy of the grain boundary can be reduced—this is one of the reasons. Another reason is that the precipitation of relative dislocations and grain boundary slippage has obvious hindrance, thereby stabilizing the grain morphology. This is also why the grain size in Figure 5 remains stable.

### 3.2. Evolution of Laves Phase During Aging

**Figure 8:**
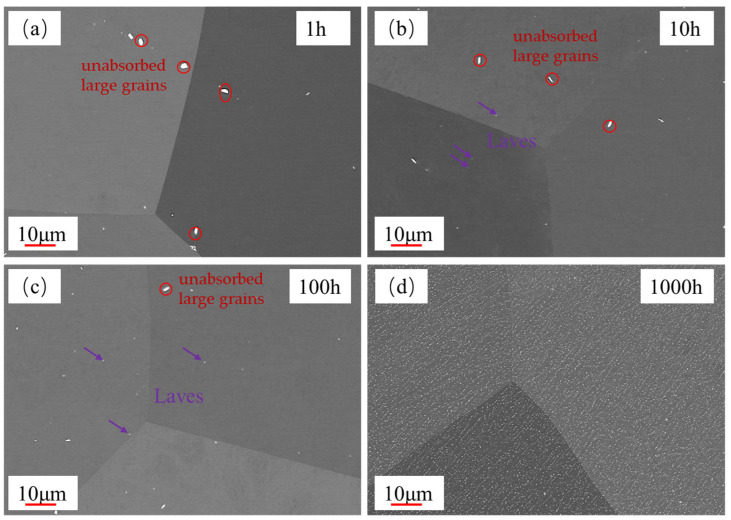
SEM images of aged specimens: (**a**) 1 h, (**b**) 10 h, (**c**) 100 h, and (**d**) 1000 h.

The SEM micrographs of different aging times are shown in Figure 8. In the red circle, unabsorbed large grains can be observed that were produced by the material during the solid solution process, and the primary precipitate is not completely dissolved. The grains were redissolved under the high temperature with increased aging. At an aging time of 10 h (Figure 8b), precipitates are observed within the grain, possibly identified as Fe_2_Nb, also known as the Laves phase. As the aging time further increases, the quantity of the Laves phase precipitates continues to rise. In particular, after aging for 1000 h, the Laves phase is uniformly distributed on both the grain and grain boundary surfaces (Figure 8d).

**Figure 9:**
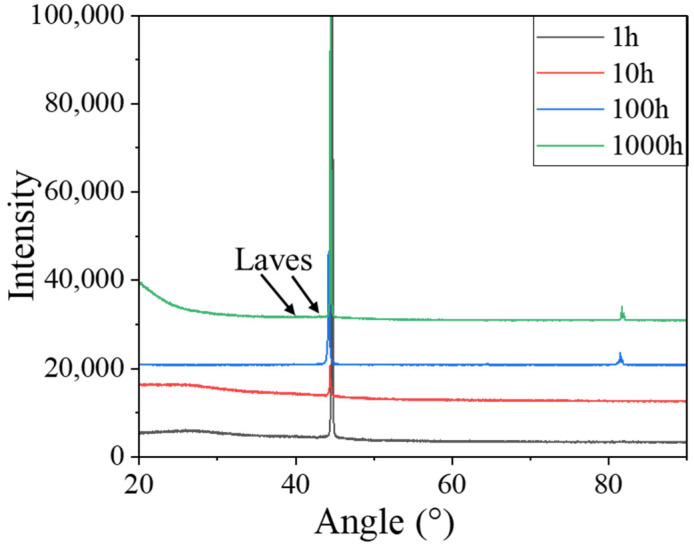
XRD patterns of specimens after aging for 1 h, 10 h, 100 h, and 1000 h.

Figure 9 shows the XRD diffraction patterns of specimens after aging at 600 °C. No peaks corresponding to Fe_2_Nb are observed in the XRD patterns of the 0.3Nb FeCrAl alloy. This absence of peaks can be attributed to the relatively high concentration of the base material in the second phase and the low content of the Nb component. Similar observations have been made by Chen et al., who detected the presence of the Fe_2_Nb phase in XRD patterns with Nb contents exceeding 1.0 wt.% [20].

The test results of the EDS of the specimen are shown in Figure 7. The primary constituents of the specimen include Fe, Nb, and Mo, along with minor amounts of Si and Ta. The contents of various components are shown in Table 2. The content of Nb is about 0.32 wt.%, which is consistent with the material composition required by the specimen. Figure 7b shows the EDS result of the precipitate indicated in Figure 7a. It may be that when EDS is complete, the analysis software usually automatically assigns the color, and the color assigned to the Fe element is black, but the display color of the elements in the software is not changed during this process, so the picture is black. Also, the greater the contents of Fe, the darker the appearance; the lesser the amount of Fe, the brighter the appearance. Therefore, it can be seen that the composition of the precipitate is Fe_2_Nb, more specifically identified as the Laves phase.

### 3.3. Tensile Properties After Aging

**Figure 10:**
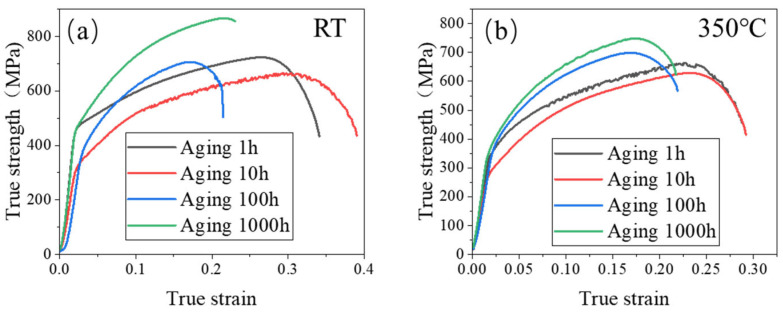
True stress–strain at (**a**) RT and (**b**) 350 °C.

The true stress–strain curves of the 0.3Nb FeCrAl alloy at RT and 350 °C tensile temperatures are presented in Figure 10. It is observed that the tensile strength initially decreases and then increases as the aging time at the same tensile temperature increases. Notably, the maximum tensile strength is achieved after aging for 1000 h. This observation is further supported by the microstructure analysis, which reveals an increase and refinement of the precipitate, leading to a more uniform distribution. Consequently, these changes in the microstructure contribute significantly to the strengthening effect observed in the 0.3Nb FeCrAl alloy. Additionally, it is observed that the slope of the true stress–strain curve remains constant with increasing aging time, indicating an unchanged work hardening rate for the alloy.

**Figure 11:**
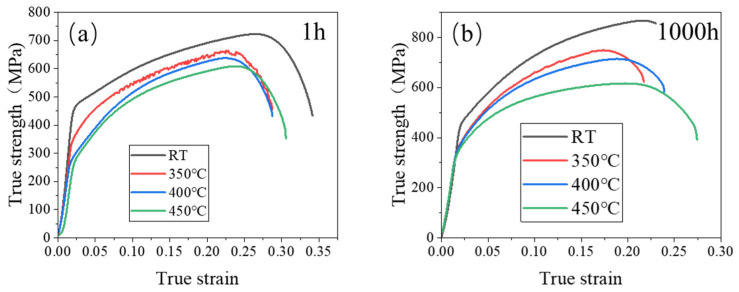
True stress–strain at aging (**a**) 1 h and (**b**) 1000 h.

The effects of different tensile temperatures on the tensile strength of 0.3NbFeCrAl alloy are shown in Figure 11. It shows that the tensile strength of 0.3Nb FeCrAl alloy gradually decreases with the increase in tensile temperature, and the tensile strength is the highest at RT. Evidently, the elevated temperature does not contribute to the enhancement of the 0.3NbFeCrAl alloy’s strength. By considering the microstructural aspects, elevating the tensile temperature facilitates the improvement of the material’s suitability for high-temperature applications, as it enhances the plastic properties of the tested specimen.

**Figure 12:**
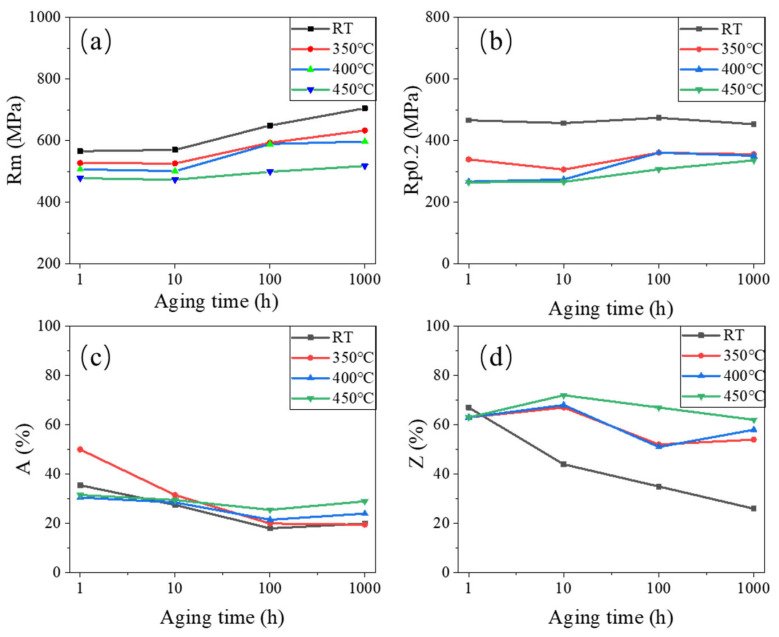
Tensile properties of aged specimens. (**a**) Tensile strength (R_m_). (**b**) Yield strength (R_p0.2_). (**c**) Elongation after fracture (A). (**d**) Section shrinkage (Z).

Figure 12 shows various tensile properties of 0.3Nb FeCrAl alloy. At constant aging time, the tensile strength (R_m_) gradually decreases with the increase in the tensile temperature. Conversely, at a constant tensile temperature, R_m_ gradually increases with the increase in the aging time. This observation indicates that aging time contributes positively to the enhancement of the tensile strength of the 0.3Nb FeCrAl alloy. However, elevating the tensile temperature does not benefit the improvement in tensile strength, as depicted in Figure 12a, where the yield strength (R_p0.2_) is seen to decrease. Conversely, increasing the tensile temperature is advantageous for improving the plastic deformation ability of the 0.3Nb FeCrAl alloy. At the given tensile temperature, R_p0.2_ increases with the growth of aging time, as shown in Figure 12b.

The percentage elongation (A) is obtained by subtracting the original gauge distance A_0_ from the post-break gauge length A_1_ and dividing it by the original gauge distance A_0_, then multiplied by 100%, as shown in Equation (1).

A = [(A_1_ − A_0_)/A_0_] × 100% (1)

As shown in Figure 12c, A exhibits a reduction with aging time before reaching 100 h. However, after aging for 100 h, A demonstrates an increasing trend with increasing tensile temperature.

The section shrinkage rate (Z) is obtained by subtracting the area Z_U_ from the original area Z_0_ and dividing it by the original area Z_0_, then multiplied by 100%, as shown in Equation (2).

Z = [(Z_0_ − Z_U_)/Z_0_] × 100% (2)

**Figure 13:**
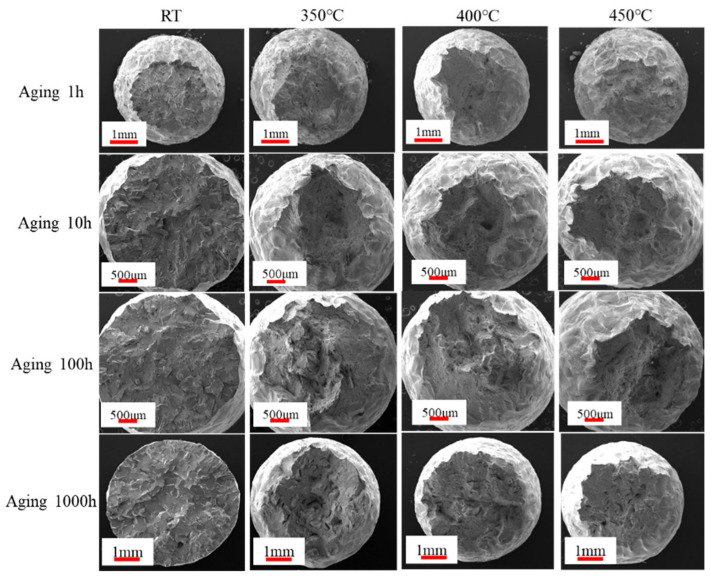
SEM images of tensile fracture at different aging time and tensile temperatures.

**Figure 14:**
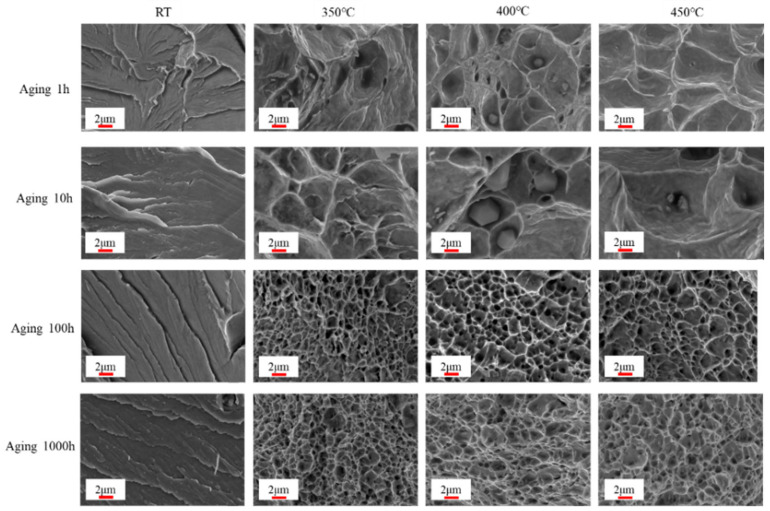
Enlarged SEM images of tensile fracture at different aging times and tensile temperatures.

Figure 12d depicts the rapid decrease in the section shrinkage rate (Z) with increasing aging time during tensile at room temperature (RT). This correlation aligns with the observed tensile section and microstructure in Figure 13 and Figure 14, suggesting that brittle fracture occurs during tensile at RT. Post-aging for 10 h, Z gradually increases with the rise in tensile temperature. This observation implies that the plastic properties of the specimens improve with increasing tensile temperature. This finding is supported by the enlarged SEM images of tensile fracture shown in Figure 14.

Figure 13 illustrates the micrograph of tensile fracture at different aging times and tensile temperatures. At RT, the extent of necking decreased with aging time, indicating that the specimen fractured shortly after the occurrence of tensile instability. This observation correlates with the shrinkage of the specimen in section Z, as shown in Figure 12. Notably, all aging conditions resulted in brittle fractures at RT, as illustrated in Figure 14. However, after aging for 10 h, the brittle fracture characteristics remained relatively unchanged with increasing aging time and tensile temperature.

**Figure 15:**
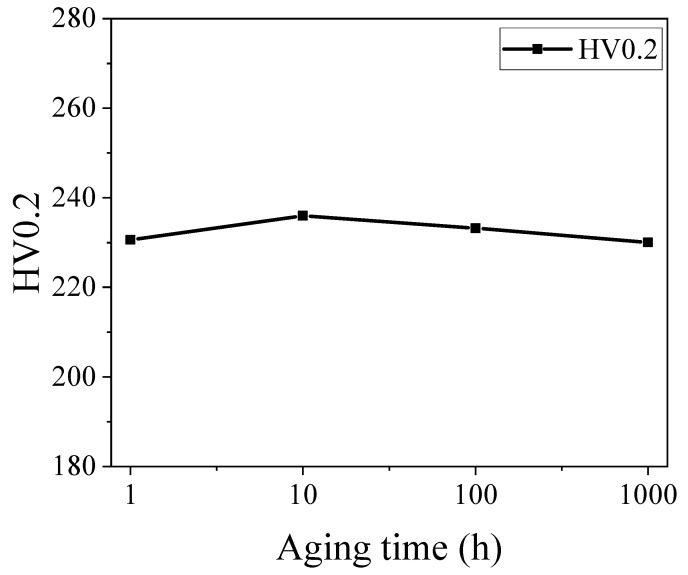
Micro-hardness of aged specimens.

**Figure 16:**
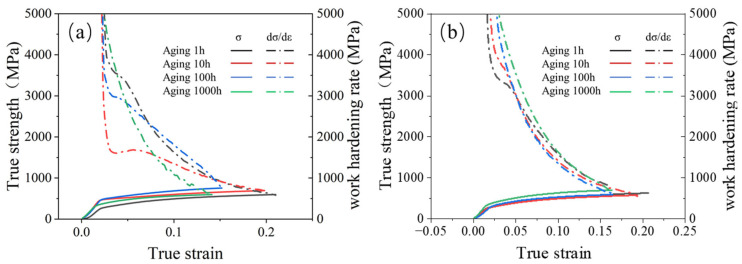
True strength corresponding to different strains and work hardening rate at (**a**) RT, (**b**) 400 °C.

Figure 15 depicts the micro-hardness behavior of the aged specimens. It is observed that the micro-hardness gradually rises as the aging time increases. Nonetheless, the degree of change is minimal owing to the favorable correlation between the work hardening rates and micro-hardness. In Figure 16, the work hardening rate exhibits a general inclination to increase with prolonged aging time. This rise may be attributed to the growing presence of precipitates. However, despite this development, the overall work hardening rate remains relatively stable. This phenomenon can be attributed to the joint influence of the precipitates and the main phase of FeCrAl alloy (namely ferrite phase).

## 4. Conclusions

The present study draws the following conclusions:

(1) It was observed that the grain size of the 0.3Nb FeCrAl alloy remained relatively stable with increasing aging time. This suggests that the alloy’s grain size is not significantly affected by aging. However, the presence of Laves phase precipitates increased gradually with aging time, and these precipitates were evenly distributed within the crystals and along the crystal boundaries;
(2) The tensile strength of the 0.3Nb FeCrAl alloy gradually increased with aging time at a constant tensile temperature. This indicates that aging can effectively improve the alloy’s tensile strength. Additionally, a higher amount of Laves phase precipitates resulted in finer grains and enhanced precipitation strengthening. Furthermore, as the tensile temperature increased, the tensile strength of the 0.3Nb FeCrAl alloy gradually decreased. Notably, the highest tensile strength was achieved at room temperature (RT);
(3) The yield strength Rp0.2 of the 0.3Nb FeCrAl alloy tended to increase with aging time, indicating the presence of aging hardening. However, at the same aging time, the yield strength displayed a decreasing trend with increasing tensile temperature. This may be attributed to the softening of the ferrite phase. The rise in tensile temperature could lead to the softening of the main phase structure of FeCrAl alloy, resulting in a reduction in yield strength;
(4) After aging for 100 h, both the A and Z gradually increased with higher tensile temperatures. This finding indicates that the plastic properties of the 0.3Nb FeCrAl alloy improved as the tensile temperature increased.

It can be seen from the above conclusions that the mechanical properties and microstructure of 0.3Nb FeCrAl alloy have hardly changed significantly during the long-term aging process under high temperature. This fully indicates that the alloy has good thermal stability and lays a solid foundation for its wide application in the industrial field in the future.

## Figures and Tables

**Table 1:** Chemical composition of 0.3Nb Fe-Cr-Al (wt.%).

Cu	Si	Mn	Nb	Mo	Al	Fe	Ti	Cr	Ni
0.0146	0.0135	0.0091	0.3271	1.9598	5.3026	81.3614	0.1122	10.2866	0.1957

**Table 2:** The content of major elements of the main phase of FeCrAl alloy after aging (wt.%).

Aging Time/h	Al	Si	Cr	Fe	Nb	Mo
1	4.31	0.35	9.88	79.84	0.32	1.98
10	4.53	0.34	9.80	79.27	0.33	2.02
100	4.51	0.35	9.85	79.23	0.32	2.06
1000	4.43	0.36	9.91	79.36	0.34	2.05

## Data Availability

The original contributions presented in this study are included in the article. Further inquiries can be directly to the corresponding authors.
